# A novel method to promote physical activity among older adults in residential care: an exploratory field study on implicit social norms

**DOI:** 10.1186/s12877-016-0394-z

**Published:** 2017-01-06

**Authors:** Margot A. Koeneman, Astrid Chorus, Marijke Hopman-Rock, Mai J. M. Chinapaw

**Affiliations:** 1 Body@Work, Research Center for Physical Activity, Work and Health, TNO-VU University Medical Center, Amsterdam, The Netherlands; 2 Department of Public and Occupational Health, EMGO Institute for Health and Care Research, VU University Medical Center, PO Box 7057, 1007 MB Amsterdam, The Netherlands; 3 TNO Netherlands organisation for applied scientific research, TNO PO Box 3005, 2301 DA Leiden, The Netherlands

**Keywords:** Aging, Automaticity, Prevention, Dependency, Disability

## Abstract

**Background:**

Physical activity (PA) levels of older adults living in a care setting are known to be very low. This is a significant health(care) problem, as regular PA has many health benefits also at advanced age. Research on automatic processes underlying PA behaviour in physically inactive older adults is yet non-existing. Since people are unconsciously influenced by people around them (i.e. by ‘social norms’) automatic processes could be used to promote PA. We developed an explorative intervention method to assess the effects of automatically processed (implicit) descriptive social norms (‘What most people do’) on behavioral intention and participation in PA offered in a local residential care setting.

**Methods:**

Forty-seven care clients met the inclusion criteria. Participants (response 45%; unaware of the intention of the research) were randomly assigned to an experimental (*N* = 10) or a control group (*N* = 11). The experimental group was exposed to photos and text heading on active peers (physically active implicit descriptive norm) using a draft newsletter article they were asked to comment on, whereas the control group was exposed to a newsletter with photos and text heading of inactive peers (physically inactive implicit descriptive norm). Subsequently, we tested (Fishers exact *p* < 0.10) whether this unaware exposure predicted intention (implicit and explicit) to participate in PA offered and organized by the care center (e.g. walking, gymnastics) and self-reported participation in organised PA at three months follow-up. Participants were debriefed later.

**Results:**

Mean age was 87 years (SD = 3.6; range 80–95) and 53% of the participants were male. At baseline, there were no significant differences in self-rated health and PA between the experimental and control group. Results indicated that implicit descriptive norm information was associated with implicit PA intention (*p* = .056, Fisher’s exact test). No significant effects were found on explicit intention. At 3 months follow-up the experimental group self-reported 80% participation in PA versus 22% in the control group (Fisher’s exact test *p* = 0.027).

**Conclusion:**

Implicit descriptive social norm information could indeed be a potentially effective way to encourage inactive older adults in residential care to engage in organized PA.

## Background

Most modern governments stimulate older adults to continue living independently for as long as possible. With the help of adapted housing, nurses, family, friends and neighbours older adults are stimulated to take care of themselves and to continue to participate in society. Although recommendations to improve physical activity among older adults exist [1], physical inactivity and sarcopenia (muscle loss) are still highly prevalent and a major health risk [2, 3] also associated with more disability [4] and higher health care costs [5, 6]. Older adults living in residential care seem to have extremely low physical activity (PA) levels, sometimes less than five minutes a day [7, 8]. This demonstrates that effective interventions for improvement of PA in care settings are highly necessary.

Until now, almost all PA promotion strategies and programs in older adults have been based on theoretical models of cognitive decision making such as the theory of planned behavior [9–13]. However, as the brain ages cognitive decision making loses its efficiency [14]. As older adults’ cognitive decision making is slowed down and disrupted, automatic processes become more influential [14–18]. Moreover, as the declining cognitive processes are unable to inhibit automatic processes, there is an even greater salience of automatic processes in decision making [19]. One of the automatic processes underlying behavior is seeing or knowing what people around us do, i.e. ‘social norms’ [20–23].

Social norms are important to regulate behavior within larger as well as smaller groups. These norms can be perceived consciously but often we are influenced by people around us without even being aware of it [24]. Nolan et al. [22] showed that for social norm information (in their study pro-environmental behavior) to affect behavior it was not required to see other people act a certain way, it was sufficient to provide norm information alone. Moreover, people were unaware of the strong influence of this norm information. Recent research has started to assess the potential role of norm information in promoting PA. Cialdini, Reno & Kallgren [21] have categorized social norms into injunctive norms, ‘what ought to be done’ and ‘what most people do’, the so-called descriptive norms. Recent research has started to assess the potential role of these descriptive norms information to promote PA. Priebe & Spink [25] for instance, utilized descriptive social norms to manipulate PA in office workers. Exposure to an e-mail message containing norm information, ‘be active because others are’, was associated with a self-reported increase in PA. Their study focused on cognitive processes and reasoned decision making. PA intention can also be influenced by *implicit* social norms [25]. Implicit social norms were for instance used to investigate whether descriptive norm information influenced intention to take up yoga [24]. Crucially, participants were unaware of the true study purpose, and were led to belief they were only judging yoga-websites designed by students. The association between descriptive norm information and behavioral intentions was moderated by perceived benefits: in the condition describing high benefits of yoga, this positive descriptive norm information indeed led to stronger exercise intentions.

The aim of our study was to assess the effect of implicit descriptive social norms on PA intention and actual participation in offered PA interventions (at follow-up) among currently physically inactive older adults in a residential care setting. We conducted an exploratory field study in which it was hypothesized that exposure to implicit social norms of active peers, was associated with implicit intention to and participation in organized PA.

## Methods

### Participants and design

Following standard procedure for psychological experiments without a medical intervention, study approval was obtained from The Medical Ethics Committee of the VU University Medical Center, Amsterdam, The Netherlands. Participants were residents of a ‘residential care centre’ in a middle-sized town in the Netherlands, and lived intramural (in the care center) or extramural (in a neighbouring home), making use of assisted living facilities provided by the residential care center. The general manager fully cooperated and supported recruitment of participants.

Participants‘ inclusion criteria were; 1) currently not participating in any of the physical activities (in a daily or weekly routine) offered by the care centre (e.g. walking, gymnastics: light aerobic exercises, mainly muscle strengthening exercise and exercises aimed at improving coordination, or dancing); 2) free of cognitive and visual impairments according to caring staff; and 3) not bedridden. Care center staff responsible for everyday care and health monitoring verified whether residents fulfilled the inclusion criteria. Eligible residents received an information letter describing the study protocol and procedure. Since we assessed the influence of automatically processed norm information it was essential participants were unaware of our study goal. The letter explained the study protocol and procedure, but left the true purpose of the study concealed. Crucially, residents remained unaware of the true study purpose, and were led to belief the study was designed to assess potential participation in routine general activities (including PA activities) organized by the care centre. The participants were further instructed not to discuss the study or its content with other residents of the care center. With the exception of general manager and head nursing, care center staff was also unaware of the true study purpose and content and were instructed to provide care as usual. All data collected was processed anonymously by assigning a participant number at the start of the experiment. Participants received a small gift as a token of appreciation.

In total 35 intramural residents (total of care clients was *N* = 159) and 12 extramural residents (from unknown number of care receiving clients outside the central building) met the inclusion criteria and received an invitation letter by regular mail to take part in the study. Ten residents directly expressed their interest in participating (by returning the written consent form). After a personal invitation from the general manager of the care center an additional 11 residents volunteered to participate and were included. In total 21 participants (intramural *N* = 19 (response = 54%), extramural *N* = 2 (response = 17%) were included in the study. A computer-generated (Matlab) randomized block design was used to equally assign male and female participants as well as those living intramural or extramural to both conditions (experimental group, *N* = 10, control group, *N* = 11). All included participants completed the first part of the study. Two participants in the control group (intramural *N* = 1, extramural *N* = 1), were not able to complete the follow-up measures because they were physically too weak at that time. These cases were excluded from the analyses concerning the follow-up data.

### Procedures

A number of steps were taken to improve the validity and feasibility of the study. The first step consisted of the researcher visiting the participants in their own home where the participants in both conditions were exposed to implicit descriptive norm information in a seemingly unrelated task (the “cover story”). The cover story was a short written news report including two photos. Participants in the experimental condition were shown the heading of the news report “increasing numbers of exercising older adults in the local community” and were asked to choose one of two photos showing older adults engaging in physical activities to include in the news report. The news report’s heading and the photos contained the descriptive norm information, conveying the active norm of other older adults (peers) being physically active. Participants in the control group were shown the heading of the news report “older adults in the local community” and were asked to choose one of two photos showing older peers (not physically active) to include in the news report. The news report’s heading and the photos contained the descriptive norm information, conveying the inactive norm of other older adults being physically inactive. Big letter types and pictures were used to facilitate ease of use.

Subsequently (second step), the researcher left the room/apartment or sat elsewhere in the apartment while the participants completed a questionnaire on implicit and explicit intention to engage in PA offered by the care center (see below). After finishing the questionnaire the researcher asked (third step) whether the participants thought their answers to the questionnaire had been influenced by anything other than their own opinion, in order to immediately assess whether the participant was consciously aware of the influence of the norm information, or other influences present, on their answers to the questions in the questionnaire.

In this study it was not possible to have a test or questionnaire before the intervention because this would be very odd to the participants not aware of any intervention.

Three months after the initial questionnaire participants received a follow-up questionnaire on actual participation in the organized activities in the residential care center, in their regular mail. The aim of this questionnaire was to show possible effects of the earlier exposure to implicit descriptive norm information on self-reported PA (operationalized as participation in the offered interventions). Finally, after four months participants were debriefed in a group session during which the general manager of the care center presented the real study aims and results.

### Outcome measures

Participants completed a questionnaire on implicit and explicit intention to engage in PA offered by the center. As no existing questionnaires were available, we designed these questions our self. The questions tap in to a variety of conscious and automatic motivations and intentions and were designed to assess the influence of descriptive norm information without revealing the true nature of the experiment. The questions in this questionnaire are described below:

*Personal relevance of the physical activities organized in the care center*. All general activities (*N* = 17 e.g. bingo, walking, library), including six physical activities that were normally organised in the care center, were listed in alphabetical order and participants were asked to indicate for each of these activities, by answering yes or no, whether it was relevant for them personally.

*Likelihood of using the physical activities organized in the care center (=implicit intention)*. All general activities, including six physical activities, were listed in alphabetical order and participants were asked to express the likelihood they would participate within the following 6 months (likely, not likely).

*Suggestion for a new activity* was asked in an open question to retrieve the potential need or suggestion of additional activities they would like to participate in. We categorized these answers into ‘physically active suggestion’ and ‘physically inactive suggestion’, following the initial categorization of the existing activities organized by the care center.

*Explicit intention to become more physically active within the following six months* was assessed using one question using a 4-point scale, ranging from ‘no intention to become more physically active’, ‘already active enough’, ‘intention to become more physically active within six months’ to ‘intention to become more physically active within one month’.

*Self-reported participation in (physical) activities provided by the care center* was assessed 3 months after the initial assessment. Again all activities in general (*N* = 17 e.g. bingo, walking, library), including six physical activities that were normally organised in the care center were listed in alphabetical order. One new physical activity was added, namely gardening club and/or garden fitness equipment. The garden activities were introduced after the initial assessment and prior to the follow-up assessment and were accessible to all participants. Participants were asked to express their participation (yes or no) within the 3 months following the initial questionnaire. Participants were asked to return the follow-up questionnaires at the mailbox at the reception of the care center.

*Basic demographic information* was collected including age, gender and educational level. Educational level was assessed on a 6-point scale ranging from ‘elementary education’ to ‘university education’ (We followed Deeg, van Tilburg, Smit & de Leeuw [26] in the construction of this question).

*Self-rated general physical health* was assessed on a 5-point scale ranging from ‘excellent’ to ‘very poor’.

*Self-rated general physical health compared to peers* was assessed on a 5-point scale ranging from ‘much better’ to ‘much worse’.

*Self-rated PA* was assessed on a 5-point scale ranging from ‘very active’ to ‘not active at all’ (We followed Deeg, van Tilburg, Smit & de Leeuw [26]) in the construction of this question).

*Self-rated PA compared to peers* was assessed on a 5-point scale ranging from ‘much more active’ to ‘a lot less active’ (adapted from Ryckman, Robbins, Thornton & Cantrell [27], *Own perception of abilities* was assessed with a question developed by the care center to assess ‘quality of life’ of the residents and was adopted in this study. This allowed for an on-invasive and familiar way to assess quality of life (i.e. ‘Is it possible for you to do what you want to do?’) and was assessed on a 4-point scale (never, sometimes, most of the time and always).

*Physical limitations* were assessed with the Dutch version of the OESO questionnaire [28]. This is a questionnaire that measures physical limitations and consists of 7 items –e.g. impaired hearing or sight and limitations in muscle strength, balance and mobility - assessed on a 4-point scale (not limited, somewhat limited, highly limited, and unable). The scores ‘highly limited’ or ‘unable’ qualified as a physical limitation. These scores were summed (range 0–7) with higher scores indicating a higher degree of physical limitations [29].

### Analyses

To check the randomization, we compared descriptive characteristics between the control and experimental group using the independent *t*-test for the variables age and physical functioning. Also, Fisher’s exact test was used to test the differences between the control and experimental group for the variables gender, educational level, perception of possibilities, self-rated general physical health, self-rated general physical health compared to peers, self-rated physical activity, and self-rated physical activity compared to peers at the start of the study. All significance levels for these tests were set at 0.05.

To test the effects of implicit descriptive norm information on PA intention we used Fisher’s exact test comparing the experimental and control condition. Because of the explorative nature of this study, significance levels were set at 0.10 (two-sided).

## Results

We first assessed whether participants were aware of the potential influence of the descriptive social norm information they were exposed to. None of the participants mentioned that their answers to the questionnaire had been influenced by anything other than their own opinion (i.e. the cover story conveying the norm information). Some of the participants experienced difficulty with writing and asked the researcher to write down their answers.

Table 1 presents participants’ physical functioning and demographic characteristics. We found no significant differences as expected after randomisation. The average age was 87 years (range: 80–95). The majority was educated beyond elementary school. Even though 42% of the participants rated their general physical health “less than good”, they also perceived themselves in “better health” than their peers (86%). The majority (86%) considered themselves fairly active and more active than their peers (76%). On average participants reported 1.3 physical limitations. The most often reported limitation (*n* = 10) was inability to walk for 400 m without stopping, even with the help of a walking aid.

**Table 1:** Baseline demographic and health characteristics of the control and experimental group

	Control	Experimental
	(*N* = 11)	(*N* = 10)
Age^a^	88.6 (3.5)	86.3 (3.6)
Male gender^b^	6 (55%)	5 (50%)
Educational level^b^		
Elementary education	3 (27%)	2 (20%)
Lower vocational education	7 (64%)	3 (30%)
General secondary education	1 (18%)	1 (10%)
Intermediate vocational education	0 (0%)	0 (0%)
Tertiary education	0 (0%)	3 (30%)
University education	0 (0%)	1 (10%)
Self-rated health^b^		
Excellent	2 (18%)	1 (10%)
Good	5 (45%)	4 (40%)
Fair	4 (36%)	3 (30%)
Poor	0 (0%)	0 (0%)
Very poor	0 (0%)	0 (0%)
Self-rated health compared to peers^b^		
Much better	5 (45%)	4 (40%)
A little better	4 (36%)	5 (50%)
Equally good/bad	2 (18%)	0 (0%)
A little worse	0 (0%)	0 (0%)
Much worse	0 (0%)	1 (10%)
Self-rated PA^b^		
Very active	4 (36%)	3 (30%)
Somewhat active	5 (45%)	5 (50%)
Not active/not inactive	2 (18%)	0 (%)
Not active	0 (0%)	1 (10%)
Not active at all	0 (0%)	1 (10%)
Self-rated PA compared to peers^b^		
Much more active	4 (36%)	4 (40%)
A little more active	4 (36%)	2 (20%)
Equally active	2 (18%)	1 (10%)
A little less active	1 (9%)	1 (10%)
Much less active	0 (0%)	2 (20%)
Perception of possibilities^b^		
Never	0 (0%)	2 (20%)
Sometimes	3 (27%)	4 (40%)
Most of the time	6 (55%)	4 (40%)
Always	2 (18%)	0 (0%)
Physical limitations (0–7)^a^	1.0 (1.3)	1.6 (1.5)

^a^Values are expressed as mean (standard deviation)

^b^Values are expressed as number (percentage)

Note. Independent *t*-test (age and physical functioning) and Fisher’s exact test revealed no significant differences between the experimental and control group

Table 2 presents the personal relevance of the (six) physical activities in the general list of 17 activities. For implicit intention, scores ranged from zero to 3 physical activities selected (out of six). In the control group four (36%) participants had the implicit intention to take part in one or more physical activities versus seven participants (70%) in the experimental group. A significant difference between the experimental and the control group was observed (*p* = 0.056, two-sided): the experimental group, exposed to descriptive norm information conveying a physically active norm indeed expressed a stronger intention to take part in physical activities organised by the care center (see Table 2 for an overview of all means and statistical tests).

**Table 2:** The differences in physical activity intention and participation between the control group and the experimental group

Outcome	Control (*N* = 11)	Experimental (*N* = 10)	*Fisher’s* *p* value of between group difference
Number of organized PA rated personal relevant^a^			ns
0	4 (36%)	3 (30%)	
1	4 (36%)	3 (30%)	
2	1 (9%)	2 (20%)	
3	2 (22%)	1 (10%)	
4	0 (0%)	1 (10%)	
Implicit intention in number of organized PA likely to use^a^			.056
0	7 (64%)	3 (30%)	
1	3 (27%)	2 (20%)	
2	0 (0%)	5 (50%)	
3	1 (9%)	0 (0%)	
Suggestion for new activity^a^			ns
Not active	7 (64%)	9 (90%)	
Active	4 (36%)	1 (10%)	
Explicit intention to take up PA^a^			ns
No, no intention	0 (0%)	0 (0%)	
No, active enough	8 (73%)	5 (50%)	
Yes within 6 months	3 (27%)	3 (30%)	
Yes within 1 month	0 (0%)	2 (20%)	
Self-reported participation in number of organized PA at three months^b^			.027
0	7 (78%)	2 (20%)	
1	1 (11%)	4 (40%)	
2	0 (0%)	3 (30%)	
3	Not reported	Not reported	
4	0 (0%)	1 (10%)	
5	1 (11%)	0 (0%)	

^a^Values are expressed as numbers (percentage)

^b^Control group *N* = 9

Participants mentioned a variety of suggestions for additional activities they would like to take part in, ranging from artistic drawing to a trip to the swimming pool. The experimental group did not propose more PA related activities compared to the control group. Nor were there significant differences between the groups for explicit PA intention. Self-reported participation in organized PA after 3 months ranged from zero to 5 activities. In the control group 2 (22%) participants had taken part in one or more PA related activities versus 8 (80%) participants in the experimental group (Fisher’s exact test, *p* = 0.027, two-sided). The experimental group did not only intend to take up more PA related activities, the self-reported participation indicated that they also took part in more PA related physical activities at follow-up (see Table 2).

## Discussion

We conducted an innovative explorative intervention study assessing the effect of automatically processed descriptive norm information on PA intention among currently physically inactive older adults in a residential care setting. We feel it is of great importance to increase the levels of PA of older adults to prevent further health and function loss and immobility. Effects of existing interventions, often based on cognitive decision making are generally disappointing. Automatic processes using descriptive norm information could potentially be an easy way of raising physical activity levels in care settings.

We found a positive statistically significant effect of providing implicit descriptive norm information on the implicit intention of older adults to engage in PA related activities, but not on their explicit intention. This was expected, as participants were unaware of their exposure to the implicit social norm. This finding is in line with the suggestion of Wiers, Minke, Kordts, Houben & Strack [30]) that the role of cues can work without conscious awareness. Thus to positively influence PA intention and participation the suggestion to become more physically active does not need to be cognitively processed, nor does the intention need to enter a conscious decision making process to influence subsequent behaviour. Future research needs to confirm this potential implicit pathway, possibly bypassing cognitive decision-making, promoting PA behaviour.

Furthermore, we found a significant positive effect of implicit descriptive norm information on self-reported participation in physical activities after 3 months, suggesting the effects were maintained.

To the best of our knowledge, our study was the first to assess the effect of implicit descriptive norm information on inactive older adults’ PA intention, after Rimal and colleagues [24] used almost the same method in college students. Our findings are in line with Rimal et al. [24], namely that priming on descriptive norm information can stimulate PA intention. Overall our study clearly showed that it was not only possible to study automatic processes in older adults, it also showed promising and lasting results. The experimental setup of exposing participants in their natural setting to implicit descriptive norm information was applied successfully, making our findings more generalizable to daily life than a lab setting. In addition, we were able to use outcome measures, such as participation in activities, to assess PA intention which was a measure relevant to our population and setting without alerting the participants on the goal of our study. Because of this alerting problem, we could not make further assessments on PA behaviour, which was of course a limitation. Future research could explore the use of more standardised outcome measures of implicit intention to allow comparison between studies. Additionally, objective measures of PA are also recommended. As explained earlier, we did not measure PA immediately before and after the intervention, because this would had the risk to unveil the unconscious cues.

In the current study we used written information (the heading of the news report) together with information conveyed by photos. Our results provide no information on the specific influence of the written information or the visual information (photo) depicting descriptive norm information. Future intervention research applying automatic processes would therefore benefit from assessing and validating various forms of conveying implicit norm information in order to find the most effective means. Additionally, assessing the amount of exposure to this information needed to successfully change norms needs to be investigated in order to maximize the efficiency of the used method.

A number of limitations need to be mentioned. First, the sample size was relatively small as this was an explorative study. Two (10%) of the participants in the control group dropped out in the follow-up. However, in the first part of the study no differences (between control and experimental subjects) in perceived possibility for participating in activities was measured. Second, participants in the experimental group were slightly higher educated. In the context of automatic versus cognitive processes it is not unlikely that educational level influences the motivation to process PA information more deliberately compared to automatic processing of this information (e.g. Elaboration likelihood model [31]). Nevertheless, the results of the present study are encouraging and shed a new light on PA promotion using implicit behavioural change techniques.

## Conclusions

In conclusion, implicit descriptive norm information could be a potentially effective way of promoting PA among inactive older adults in residential care. Therefore, research into application of implicit behavioural change techniques in PA interventions deserves more attention.

## Abbreviations

OESO: (in English OECD = Organisation for Economic Co-operation and Development)
PA: Physical activity
SD: Standard deviation
